# Dietary Plant-Based Mixture Improves Feed Efficiency, Gross Profit, Physiological Performance, Gene Expression and Gut Health of Nile Tilapia (*Oreochromis niloticus*)

**DOI:** 10.3390/biology14020186

**Published:** 2025-02-11

**Authors:** Abdel-Fattah M. El-Sayed, Mahougnon Simeon Fagnon, Amira M. Hamdan, Thibaut Chabrillat, Sylvain Kerros, Salma M. S. Zeid

**Affiliations:** 1 Oceanography Department, Faculty of Science, Alexandria University, Alexandria 21526, Egypt; amira_hamdan1978@yahoo.com (A.M.H.); salmazeid2007@gmail.com (S.M.S.Z.); 2 Phytosynthese, 57 Avenue Jean Jaurès, 63200 Mozac, France; thibaut.chabrillat@phytosynthese.fr (T.C.); sylvain.kerros@phytosynthese.fr (S.K.)

**Keywords:** green propolis, immune and antioxidant response, Nile tilapia, Phyto AquaNity, standardized botanical specialty, turmeric

## Abstract

The goal of this study was to evaluate the effect of the supplemental plant-based blend Phyto AquaNity (PAN) on growth, profitability and health status of Nile tilapia (*O. niloticus*) cultured in hapas. Juvenile fish of average weight 73 g were stocked in 2-m^3^ hapas in triplicates at a density of 20 fish m^−3^ (40 fish hapa^−1^). Four extruded diets were formulated following isonitrogenous (30% CP), isoenergetic (17 MJ kg^−1^) standards, with incorporation levels of 0, 0.25, 0.50, or 1.00 g of PAN kg^−1^ diet. Fish were fed the experimental diets at a daily rate of 2–3% of their body weight (BW) over 80 days. From this study, results showed that growth performance and tissue composition were not significantly impacted by these treatments. Feed intake exhibited a significant reduction while feed efficiency was improved in the groups fed 0.5 g kg^−1^. Diets supplemented with PAN were also more profitable than the control diet, with 0.5 g kg^−1^ indicating the highest return (9.24% increase). Digestive enzyme activity and hepatic enzyme activity were significantly improved with an increase in the level of PAN up to 0.50 g kg^−1^. In addition, immune parameters (lysozyme activity, alternative complement and phagocytic cells) and antioxidant enzymes (superoxide dismutase, glutathione peroxidase and catalase) were significantly enhanced with increasing PAN level up to 0.5 g kg^−1^ (*p* < 0.05). Gene expression of cytokines was also upregulated in fish fed with PAN at levels of up to 0.5 g kg^−1^ (*p* < 0.05). Moreover, the results showed that higher levels of PAN promoted the growth of beneficial bacteria while reducing the load of pathogenic bacteria. Additionally, intestinal fold length and goblet cell count were significantly increased in the group fed PAN up to 0.5 g kg^−1^. A quadratic regression analyses performed on feed efficiency, profitability, hepatic enzyme activity and gut microbial load revealed that 0.60–0.65 g kg^−1^ of feed were the optimal dosages. Regarding immunological and antioxidant responses, the optimal dosages ranged between 0.75 and 0.85 g kg^−1^. Overall, about 0.60–0.85 g PAN kg^−1^ feed is suggested for optimum performance, profitability, gut health and overall health status of Nile tilapia, depending on the target objective for which this product is added.

## Simple Summary

The present study aimed to assess the use of a dietary botanical blend (Phyto AquaNity (PAN)) as a natural feed additive for Nile tilapia grown in pond-based hapas. The incorporation of PAN into diets did not affect growth rates. PAN improved feed efficiency and profitability. This additive also enhanced digestive enzyme activity, hepatic enzyme function, and immune and antioxidant parameters. Gene expression of cytokines was significantly upregulated in fish fed with PAN. Meanwhile, dietary PAN improved gut health parameters, increased the beneficial microbial counts and decreased pathogenic bacteria in fish gut. These results emphasize the importance of supplemental PAN in modulating immune and antioxidant responses and improving the health status of Nile tilapia cultured under field conditions.

## 1. Introduction

Tilapia (family: Cichlidae) are currently among the top cultured finfish group in the world [1]. With a global production estimated at 6.3 million mt in 2021 (over USD 12 billion in terms of value), this group of species is farmed in more than 120 countries [1,2]. However, the development of tilapia cultures, along with the gradual intensification, has rendered tilapia more vulnerable to stress and infectious/opportunist pathogens, leading to mass mortality and production loss [2,3]. This situation has been associated with the overuse or misuse of antibiotics and other chemical drugs, which has raised significant health and public concerns. Public awareness about the safety and environmental impact of synthetic drugs in aquaculture has increased, highlighting the need for other safe and more sustainable alternatives.

Therefore, emphasizing the importance of enhancing the immunity and resilience of aquatic animals can reduce the dependency on antibiotics and chemical treatments [4,5]. Thus, searching for natural, eco-friendly supplementations to enhance fish immunity and resilience has become necessary. In this regard, many solutions have emerged in recent years as genuine substitutes for traditional drugs in aquaculture [6,7,8].

Among the wide range of solutions currently applied in aquaculture, botanical-based products are receiving considerable attention as natural alternatives to chemical drugs [9,10,11,12]. These plant products are separately used according to the composition of their active compounds as growth promoters, immunostimulants, antioxidants, gut health stimulators and antimicrobial and anti-parasitic agents [10,12].

Propolis and turmeric (*Curcuma longa*) are among the natural plant-based products that have been tested in tilapia culture, except standardized Brazilian green propolis. Propolis is a natural substance collected by honeybees from the different organs of plants. Specifically, Brazilian green propolis (collected from *Baccharis dracunculifolia*, an endemic plant species) is rich in Artepillin C and flavonoids. Green propolis is well known for its immunomodulatory and anti-inflammation properties in different organisms, including aquatic animals [13,14,15,16,17]. Overall, supplemental propolis was demonstrated to enhance the growth performance, immune parameters and disease resistance of Nile tilapia (*Oreochromis niloticus*) [15,18,19,20] and Mozambique tilapia (*Oreochromis mossambicus*) [21]. In addition, propolis extract improved the resilience of Nile tilapia to low-temperature challenges (winter stress) [22].

Turmeric is a rhizomatous plant belonging to the Zingiberaceae family, with curcuminoids being the most studied turmeric components. Curcuminoids are a strong antioxidant compound because it has a high membrane affinity with lipid bilayer, thereby exerting strong intracellular activities [23,24,25]. These compounds also prevent the disruption of tight junction protein organization and improve immune responses as well [25,26].

Many studies have investigated curcumin, the main curcuminoid in turmeric, as an additive in tilapia feed, with encouraging results [27]. For example, turmeric supplementation improved growth rates, disease resistance and immunostimulatory effects in Nile tilapia [28]. The authors of [29] also found that supplemental curcumin enhanced growth rates, flesh quality, immunomodulation and antioxidant activities in Nile tilapia. Likewise, [30] demonstrated that dietary curcumin improved immune responses, antioxidant property and gut microbiota balance in Nile tilapia reared at low temperatures. Similar results were also reported in red tilapia (*Oreochromis* sp.) supplemented with curcumin and challenged against *Aspergillus flavus* [3]. In addition, growth hormone, leptin and hepatic growth factors were impacted by the inclusion of curcumin in the diet of Mozambique tilapia [31].

It is evident from the above overview that Artepillin C from green propolis and curcuminoids from turmeric can separately have positive effects on tilapia species. However, the effects of a mixture of these two bioactive compounds have not been investigated, especially in Nile tilapia and in field conditions. Therefore, the present study was carried out to evaluate the effects of a commercial mixture of Artepillin C and curcuminoids (Phyto AquaNity^TM^) on growth rates, feed efficiency, profitability, digestive and hepatic enzymes activity, immune and antioxidant response, gene expression, intestinal microbiota and gut health of Nile tilapia cultured under field conditions.

## 2. Material and Methods

### 2.1. Fish and Experimental Facility

This trial was conducted using 2-m^3^ hapas of 2 m in length, 1 m in width and 1.5 m in height. These hapas were fixed in a commercial tilapia pond located at Edku, Behaira Governorate, Egypt (see [32] for details). The mesh size of the hapas was 10 mm^2^. Healthy monosex male Nile tilapia, with an initial mean weight of about 70 g per fish, were obtained from the same fish farm in which the trial was carried out. Experimental fish were stocked into the hapas at a density of 20 fish m^−3^ (40 fish per hapa) under three replicates. Fish were acclimatized with experimental diets over one week. After this period, fish in each hapa were netted, weighed and counted. The mean initial fish weight was around 73 g per fish.

### 2.2. Experimental Diets and Feeding

Four experimental extruded diets with isonitrogenous (30% CP), isocaloric (17 MJ kg^−1^) energy contents were produced. These diets contained 0 (control), 0.25, 0.50 and 1.00 g of Phyto AquaNity (PAN) kg^−1^ diet, designated as PAN_0_, PAN_0.25_, PAN_0.5_ and PAN_1_, respectively (Table 1). These inclusion ranges were based on the study previously performed on white-leg shrimp [17]. The experimental diets were manufactured by Makkah Aquafeed Mill (Kafr Elsheikh Governorate, Egypt). At the beginning of the trial, the daily feeding rate was 3% of fish body weight (BW). The diet was distributed to fish twice per day as follows: 8:00–9:00 am in the morning and 3:00–4:00 pm in the afternoon. During the trial, the feeding rate was adjusted to 2.5% when the water temperature dropped to 23–25 °C, and it was reduced to 2% when the water temperature fell below 23 °C. Fish were weighed every 10 days in order to adjust the daily amount of feed. After 80 days, the trial was ended due to the drop in water temperature to 22 °C. During the trial, the range and average (in parenthesis) of the water quality parameters measured were summarized as follows: temperature, 22.0–28 °C (25.45 °C); dissolved oxygen, 4.30–5.87 mg L^−1^ (5.54 mg L^−1^); pH, 7.6–8.45 (8.17); and NH_4_-N, 0.03–0.07 mg L^−1^ (0.053 mg L^−1^).

At the termination of the feeding trial, 24 h fasting was applied to all fish. On the harvest day, fish in each hapa were weighed and the total number in each hapa was counted to calculate the average weight. A total of 4–5 fish were sampled in each hapa and rapidly anesthetized with clove oil (0.5 mL L^−1^) for further chemical and physiological analysis.

### 2.3. Calculation of Growth and Feed Efficiency

To assess the fish growth performance and feed efficiency, the following formulas were used for the calculation:

(a) Weight Gain (WG) = Final weight (Wf) − Initial weight (Wi); expressed in g;
(b) Specific Growth Rate (SGR) = 100 × (ln Wf − ln Wi)/trial period (days); expressed in % day^−1^;
(c) Feed Conversion Ratio (FCR) = Feed Intake (FI)/Body Weight Gain (WG);
(d) Protein Efficiency Ratio (PER) = Body Weight Gain (WG)/Protein Intake (PI);
(e) Survival = 100 × (Final stocked fish number/Initial stocked fish number); expressed as a percentage (%).

### 2.4. Economic Analysis

In order to evaluate the profitability of the supplementation of PAN in the diets, economic analysis was realized. Total fish yield, fish sales, and the amount and cost of consumed feed were considered. The following parameters were calculated for this evaluation:

- Total feed intake (kg m^−3^) = FCR × weight gain m^−3^.
- Total feed cost (USD m^−3^) = amount of feed intake m^−3^ × feed price in USD kg^−1^.
- Total fish sales (USD m^−3^) = fish yield (kg m^−3^) × fish price in USD kg^−1^.
- Gross profit over feed cost (USD m^−3^) = total fish sale − total feed cost.
- Profitability over the control diet (%) = 100 (profit from fish fed PAN diets − profit from fish fed the control diet)/profit from fish fed the control diet.

### 2.5. Whole Body Composition and Flesh Quality

At the beginning of the trial, 5 fish were sampled and frozen at −20 °C for initial proximate analyses. At the end, 4–5 fish were sampled from each hapa and stored in a labeled vial at −20 °C to perform the final body analysis. This sample size allowed us to collect sufficient flesh samples from 10 to 12.5% of the fish in each hapa. Pooling the flesh of 4–5 fish would also lead to more accurate and reliable results. These analyses of tilapia flesh and experimental diets were carried out as described by [32]. Briefly, preweighed samples were dried in an oven at 100 °C at a constant temperature to determine the moisture content. Regarding the nitrogen value, it was measured using the Kjeldahl method (2200 Kjeltec Auto distillation, Foss Tecator, Hogonas, Sweden). The total protein was estimated by multiplying the nitrogen percentage by 6.25. In addition, the crude lipid content (ether extract) was determined using the solvent extraction method (1045 Soxtec Extraction Unit, Foss Tecator, Hogonas, Sweden) using diethyl ether (boiling point, 40–60 °C) as an organic solvent. To determine the ash content, a muffle furnace was used for incinerating preweighed samples at 650 °C for 6 h. The difference was considered to be the nitrogen-free extract (NFE).

### 2.6. Immunological and Antioxidant Enzyme Analysis

A total of 4–5 fish were also sampled to collect blood from their caudal veins with sterile syringes. The first group of blood samples was stored in heparinized tubes and used for whole blood analysis. The second group was kept in non-heparinized tubes and used for plasma analysis. These analyses were performed by following the described methodology of [33]. Phagocytic activity, lysozyme activity, ACH50, superoxide dismutase activity, phenoloxidase and glutathione were analyzed from plasma obtained by centrifugation (6000× *g* for 10 min) at 4 °C.

#### 2.6.1. Phagocytic Activity

Phagocytic activity (PA) was measured according to the method followed by [34]. A total of 200 mL of leucocyte suspensions were added in AL medium in triplicate tubes. These were inoculated with 100 μL of formalin-killed *Staphylococcus aureus* ATCC 25923 in phosphate-buffered saline (PBS). This strain was obtained from the Naval Medical Unit 3 in Cairo, Egypt. A centrifugation of tubes was performed at 3000× *g* for 5 min at 4 °C. Resulting pellets were applied to microscope slides and treated with Wright–Giemsa solution (Sigma-Aldrich, St. Louis, MO, USA). Phagocytic cells were counted per 100 adhered cells. PA activity was expressed as follows: PA = 100 (phagocytic leucocytes)/(total leucocytes).

#### 2.6.2. Lysozyme Activity

A total of 50 μL of serum was combined with 950 μL *Micrococcus lysodeikticus* suspension (200 mg mL^−1^) obtained from Jiancheng, Jiangsu, China. The solution was buffered with 0.05 M of sodium phosphate at pH 6.2. at 25 °C. With the spectrophotometer (UV2802S, Shimadzu, Kyoto, Japan), the optical density (OD) was recorded after 0.5 and 6 min at 530 nm. A single unit of lysozyme activity represents the quantity of serum lysozyme that reduces the absorbance by 0.001 min^−1^ at 530 nm. Lysozyme activity was expressed in U mL^−1^.

#### 2.6.3. Alternative Complement Activity

This analysis was realized following the methods described by [35]. Briefly, 0.5 mL of diluted serum was combined with 0.2 mL of suspension of rabbit erythrocyte (2 × 10^8^ cells mL^−1^). The obtained mixture was incubated at 20 °C with a pH at 7.0 for 2 h in 10 mM EGTA and 10 mM MgCl_2_. A total of 1.4 mL of gelatin veronal buffer (GVB) containing 10 mM EDTA was added after hemolytic reaction was stopped. The solution was then centrifuged, and the OD of the supernatant was measured at 414 nm. ACH50 was determined as unit mL^−1^ of the volume of serum complement required to induce 50% hemolysis.

#### 2.6.4. Antioxidant Enzymes

In this section, the methodologies used to perform the analysis of the following antioxidant enzymes are described: superoxide dismutase (SOD), phenoloxidase (PO) and glutathione peroxidase (GPx) activities with malondialdehyde (MDA).

SOD activity was evaluated using a spectrophotometer at a wavelength of 505 nm [36]. One unit of SOD activity was defined as the quantity of enzyme necessary to reduce 50% cytochrome rate in the control group.

PO activity was assessed based on the formation of chromophore from l-dihydroxyphenylalanine (L-DOPA) (Sigma–Aldrich, St. Louis, MO, USA), using a negative control for spontaneous oxidation of the L-DOPA. A single unit of enzyme activity was expressed as the increase in absorbance of 0.001 mg^−1^ protein at 490 nm.

MDA was measured calorimetrically [37]. A total of 10 μL of sampled homogenates were added to glass tubes, with thiobarbituric acid (0.8%, Sigma), acetic acid (20%), Milli Q water and sodium dodecyl sulfate (8.1%). The solution was incubated at 95 °C for 30 min. It was cooled, and n-butanol was mixed and centrifugated at 3000× *g* for 10 min at 15 °C. N-butanol phase was transferred to a white 96-well ELISA microplate. Fluorescence was measured with a fluorometer equipped with a microplate reader (Victor 2, Perkin Elmer) (Waltham, MA, USA) at an excitation wavelength of 520 nm and an emission wavelength of 580 nm. GPx activity was analyzed using the method described by [38]. Briefly, 2 mL reaction mixture was prepared with 1.49 mL of phosphate buffer (0.1 M, pH 7.4), 50 µL of glutathione reductase (1 U mL^−1^), 100 µL of EDTA (1 mM), 100 µL of sodium azide O_2_ (0.25 mM), 100 µL of NADPH (0.1 mM), 10 µL of H_2_, 50 µL of GSH (1 mM) and 100 µL of homogenate (sample). The decrease in NADPH at 340 nm was monitored at 25 °C. Enzyme activity was expressed as nM NADPH oxidized min^−1^ mg^−1^.

### 2.7. Digestive Enzymes Analysis

Samples from 4 fish intestines per hapa were aseptically collected and rinsed with cold distilled water. Their contents were homogenized using phosphate-buffered saline (PBS pH 7.5) at 4 °C. A centrifugation of the homogenates was carried out at 5000× *g* for 20 min at 4 °C. Then, the supernatant was collected and stored at 4 °C. The enzymatic analyses were performed within 24 h after extraction. Intestinal amylase, protease and lipase activities were determined according to the standard methods [39,40]. These were expressed as a specific activity (U mg^−1^ intestine content).

### 2.8. Liver Function Enzymes

A total of 3–4 fish per hapa were sampled to remove their liver organ. Enzymatic activities of the liver were analyzed following the methods described by [41]. Alanine aminotransferase (ALT) and aspartate aminotransferase (AST) activities were measured by evaluating the increase in absorbance at a specific wavelength due to the formation of pyruvate (ALT), the formation of oxaloacetate (AST) or the decrease in NADH absorbance. Lactate dehydrogenase (LDH) activity was assessed by measuring the decrease in absorbance at a specific wavelength due to the consumption of NADH or the increase in absorbance due to the formation of pyruvate [42].

### 2.9. Expression of Cytokine Genes

A total of 3 fish from each hapa (9 fish per treatment) were collected for their liver. These tissues were labeled and stored on dry ice until the sampling was completed. The samples were then kept at −80 °C until analyzed. Real-time PCR was used to assess the expression of these genes. The total RNA from liver tissues was isolated using a Tri Pure Agent (Aidlab, Beijing, China) according to the manufacturer’s protocol, and their quality and quantity were measured using a NANODROP 2000 spectrophotometer (Thermo, Waltham, MA, USA). The cDNAs of the total liver RNA were analyzed using a Prime-ScriptTM, RT reagent Kit with a gDNA Eraser (Perfect Real Time, Takara, Kusatsu, Shiga, Japan). The reference gene was β-Actin. The PCR volume was 10 μL. It consists of 1.0 μL of cDNA template, 5.0 μL of SYBR Premix Ex Taq, 1.0 μL of PCR primers (5 μM) and 3.0 μL of nuclease-free water. The following program was adopted for the qRT-PCRs: 95 °C for 30 s; 36 cycles of 95 °C for 5 s; and 60 °C for 20 s in the CFX Connect Real-Time System (Bio-Rad). The qRT-PCR primers were designed to span an intron to ensure specificity on intended genes. qPCR efficiency ranged from 98 to 102% with a correlation coefficient higher than 0.97 for each gene. the expression of tested genes was calculated with the 2^−ΔΔCt^ method. Table 2 presents the name and primer sequences of the target genes. Stability analysis was carried out to validate the housekeeping gene (β-Actin) stability, as described by [43].

### 2.10. Analysis of Gut Microbiota

Gut microbiota analysis was performed following the methods outlined by [44,45]. In brief, 4 fish per hapa were selected for intestinal analysis. Intestines were aseptically collected and mixed in 90 mL of 0.85% sodium chloride solution. The obtained mixture was strained through a nylon sterile membrane filter mesh (100 µm). Then, the homogenates were sequentially diluted to 10^−4^ using 9 mL of sterile 0.85% saline solution. For total plate count assessment, 0.1 mL of each homogenate was evenly spread onto duplicate plates of Tryptone soya agar (TSA), MRS agar and blood agar. These media were used to isolate specific viable bacteria species, including *Bacillus*, *Lactobacillus*, *Staphylococcus* and *Streptococcus*. The plates were then incubated for 72 h at 28 °C following the protocols outlined by [44,46]. Dominant colonies were isolated, purified and identified based on their morphological characteristics through Gram staining and biochemical tests (API 50CHL and 50CHB kits) (bioMérieux, Craponne, France-Catalogue number 50300) as described by the manufacturer. The bacterial cell counts were measured and expressed as CFU mL^−1^.

### 2.11. Intestinal Histological Analysis

A total of 3 fish were randomly netted from each hapa (9 fish per treatment) for intestinal histological analysis. Intestines were removed, pooled and stored in ethyl alcohol (AR-95%). Anterior, mid and posterior gut were sectioned and fixed in Bouin’s solution for 18–24 h and dehydrated in decreasing concentrations of ethanol. Sections of 4–5 μm thickness were stained with periodic acid-Schiff (PAS) for goblet cell staining and with hematoxylin and eosin for general morphometry. Intestinal villi length was measured using NIH image analysis software, V 1.61 (NIH Image, Bethesda, MD, USA).

### 2.12. Statistical Analysis

Data are presented as mean ± standard error (SE) of three hapas (n = 3). A Shapiro–Wilk test was performed to check for normality. The Shapiro–Wilk test was applied to check the normality for each variable and Levene’s test was used to assess their homogeneity. When these assumptions were validated, an ANOVA was run to evaluate the difference between all treatments at a significance level of 95% (*p* < 0.05). A Student’s *t*-test was applied to evaluate the difference between the control and PAN groups. R software (https://www.R-project.org, R version 4.2.2) was used to perform all these analyses. Simple linear and nonlinear regressions were carried out to correlate the relationships between measured parameters and dietary PAN concentrations, using the SPSS program, version 12 (SPSS Inc., Chicago, IL, USA).

## 3. Results

### 3.1. Growth Performance

**Figure 1:**
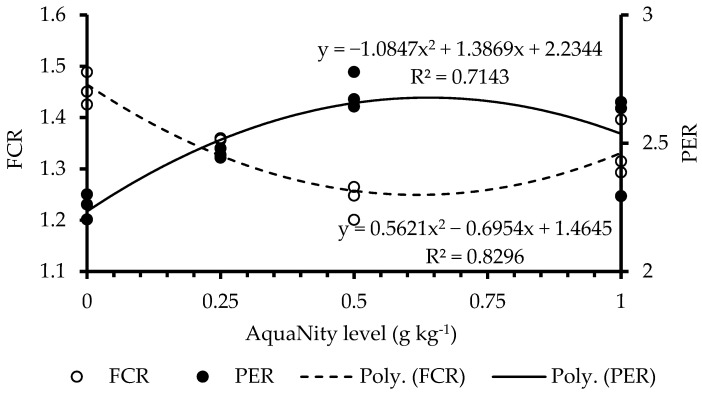
Feed conversion ratio (FCR) and protein efficiency ratio (PER) of tested Nile tilapia.

In this study, growth rates and fish survival were not significantly affected by Phyto AquaNity (*p* > 0.05) (Table 3). In fact, no mortalities were recorded throughout the trial, except that three fish jumped from three different hapas during fish sampling and weighing. One may argue that the jumped fish may have affected the final fish biomass and average fish weight. However, this may not be the case because we had two other replicates having 40 fish hapa^−1^. The average weight in the third hapa was calculated by dividing the total fish biomass by 39 fish instead of 40 fish. This simply means that if 1 fish (out of 120 fish per treatment) is lost from 1 hapa the overall average weight per fish would not be significantly affected. Fish fed on PAN-supplemented diets showed significantly lower feed consumption (*p* < 0.05) and better feed conversion ratio (FCR) and protein efficiency ratio (PER) than the control diet. Quadratic regression analysis indicated that the best FCR and PER values were determined at about 0.60 g PAN kg^−1^ feed (Figure 1).

### 3.2. Economical Analysis

Gross profits increased significantly in the groups fed with PAN (*p* < 0.05) (Table 3). A total of 0.50 g PAN kg^−1^ diet led to the highest gross profit (+9.24%) over the feed cost. At 0.25 and 1.0 g PAN kg^−1^, the profitability was 4.08 and 5.67%, respectively. Quadratic regression showed that the best profitability was also found at about 0.60 g PAN kg^−1^ feed.

### 3.3. Body Composition

Nile tilapia flesh composition (on wet weight basis) is summarized in Table 4. The results indicated that body composition was not significantly affected (*p* > 0.05) by supplemental PAN.

### 3.4. Digestive and Hepatic Enzymes

Table 5 presents the digestive enzyme activities (protease, amylase and lipase) and liver enzyme activities (LDH, AST and ALT). Protease, lipase and amylase activity significantly increased with increasing dietary PAN supplementation up to 0.50 g kg^−1^ (*p* < 0.05). From regression analysis, it was demonstrated that the maximum enzymes activity of trypsin and lipase was obtained at about 0.75 g PAN kg^−1^, while the highest amylase activity occurred at 0.85 g kg^−1^ feed.

PAN groups significantly reduced the activities of liver enzymes (AST, ALT and LDH) in comparison with the control diet (*p* < 0.05). The lowest values of AST, ALT and LDH were recorded at 0.5 g PAN kg^−1^. These values slightly increased at PAN incorporated at 1 g/kg (*p* > 0.05). The quadratic regression results showed that the lowest values of hepatic enzymes ranged between 0.60 and 0.65 g of PAN kg^−1^ feed.

### 3.5. Immunological and Antioxidant Responses

**Figure 2:**
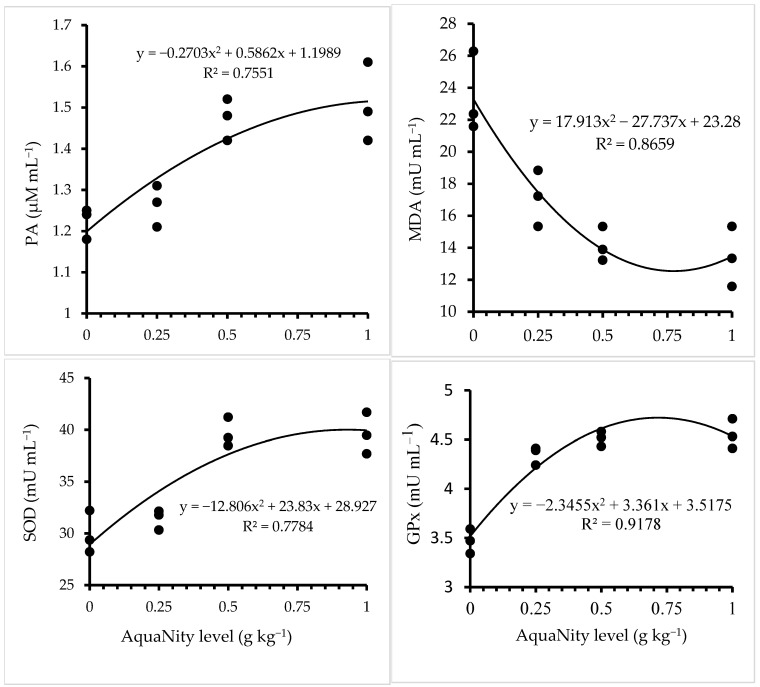
Immunological and antioxidant parameters in Nile tilapia fed the test diets. PA, phagocytic activity; MDA, malondialdehyde; SOD, superoxide dismutase; GPx, glutathione peroxidase activity.

Fish fed with the diets including PAN were significantly affected on immunological parameters (Table 6). Thus, lysozyme activity, alternative complement activity (ACH50), phagocytic activity (PA), phenoloxidase activity (PO), superoxide dismutase (SOD) and glutathione peroxidase (GPx) were all significantly increased (*p* < 0.05) with increasing dietary PAN. These values were slightly decreased in the group of PAN at 1.0 g kg^−1^. However, these values were higher at all PAN levels than in the control (0) group. Malondialdehyde (MDA) was significantly decreased with increasing dietary PAN (*p* < 0.05). The quadratic regression analysis demonstrated that the optimum immunological and antioxidant responses were achieved at 0.75–0.85 g kg^−1^ (Figure 2).

### 3.6. Cytokine Gene Expression

The expression of all studied genes was significantly upregulated in a dose-dependant manner (Table 7), and 0.5 g PAN kg^−1^ diet produced the best gene upregulation (*p* < 0.05). Beyond 0.5 g kg^−1^ of PAN, the gene expression was reduced or leveled off, except for *IL-1β*, which was significantly higher at 1.0 g kg^−1^ than at other PAN levels. The quadratic regression analysis exhibited a maximum gene expression between 0.70 and 0.80 g of PAN kg^−1^ diet.

### 3.7. Gut Microbiota

**Figure 3:**
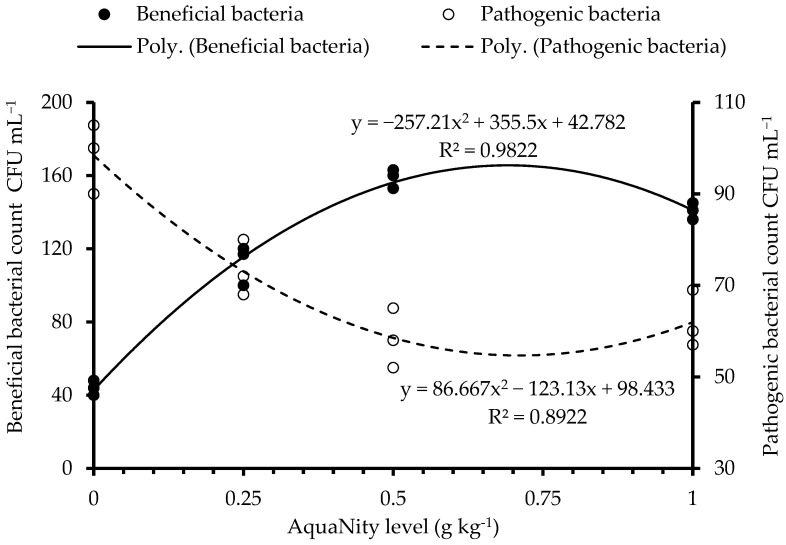
Gut microbial counts of Nile tilapia fed the experimental diets.

The present results revealed that total gut microbial counts were significantly higher in the groups fed with PAN (*p* < 0.05) than the control diet (Table 8). Bacterial counts increased in a dose-dependent manner and then decreased at PAN 1.0 g kg^−1^. However, quadratic regression analysis indicated that the maximum microbial count was achieved at 0.65 g PAN kg^−1^ diet. Regarding the pathogenic Gram-negative bacteria (*Pseudomonas*, *Vibrio* and *Salmonella*) the highest number was obtained in the control group. PAN groups significantly reduced these bacterial counts and enhanced the beneficial, mainly Gram-positive bacteria *(Bacillus* sp.) counts (*p* < 0.05). On the contrary, a significantly lower abundance of pathogenic bacteria (*Staphylococcus*; Gram-negative) was recorded in the guts of Nile tilapia fed PAN-based diets compared to the control fish group. PAN groups reduced the number of pathogenic bacteria by up to 25.4%, 40.7% and 34.6%, at 0.25, 0.50 and 1.0 g kg^−1^, respectively. Quadratic regression analysis demonstrated that the highest total viable bacterial counts, beneficial counts and lowest pathogenic bacterial counts were approximately around 0.70 g PAN kg^−1^ feed (Figure 3).

### 3.8. Intestinal Histology

**Figure 4:**
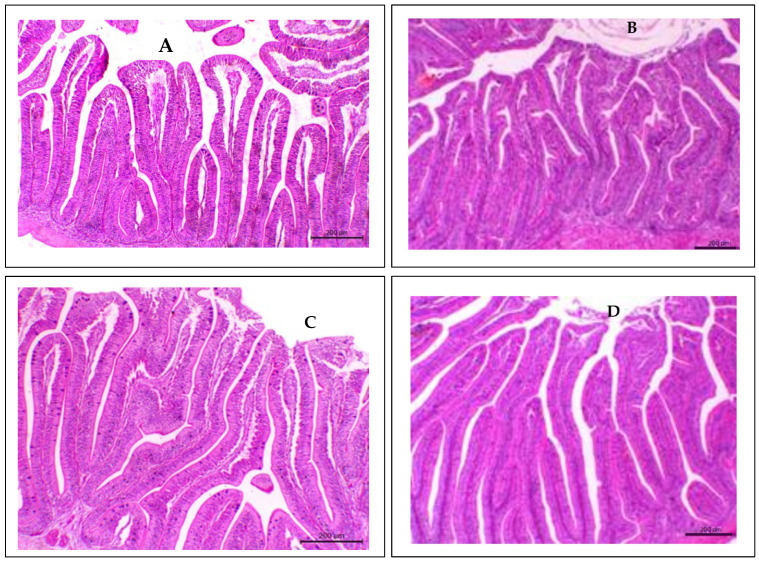
Photomicrograph of the mid-gut of *O. niloticus* fed at 0 (**A**), 0.25 (**B**), 0.5 (**C**) and 1.0 (**D**) g PAN kg^−1^ diet.

Intestinal fold length and the number of goblet cells significantly increased in the anterior, mid and posterior guts of Nile tilapia fed PAN-based diets, compared to the control diet (Table 9 and Figure 4). Meanwhile, interfold spaces showed an opposite trend. However, these parameters were significantly higher in the mid-gut than in the anterior and posterior parts of the gut (*p* < 0.05). The regression analysis showed high correlations between dietary PAN and gut structure. This analysis exhibited the highest intestinal fold length, interfold space and goblet cell count at about 0.75 g PAN kg^−1^ feed.

## 4. Discussion

The present study investigated a dietary plant-based mixture (Phyto AquaNity-PAN), including mainly Artepillin C (green propolis) and curcuminoids (turmeric), for Nile tilapia. The major bioactive product of turmeric rhizome (the most used) is the curcuminoids, which represent approximately 1.5–5% [27]. Curcuminoids comprise three main compounds, curcumin (60–70%), desmethoxycurcumin (20–27%) and bisdemethoxycurcumin curcumin (10–15%) [47]. Meanwhile, Brazilian green propolis extracts contain mainly Artepillin C, Baccharin and Galangin [16]. The tested product (PAN) is standardized and rich in these two biomolecules, with curcuminoids following the European Food Safety Authority (EFSA) recommendation of 15 ppm for fish [48].

In this study, PAN did not significantly improve the growth rates of *O. niloticus* (*p* > 0.05), although an evident trend was observed. In Genetically Improved Farmed Tilapia (GIFT) fed curcumin-based diets, similar results were also observed [49]. Growth performance parameters of Nile tilapia were also not significantly affected by supplemented propolis at 0.5, 1.0 and 1.5 g kg^−1^ [50]. Other studies revealed that dietary curcumin improved growth parameters and feed efficiency of this fish species [51,52,53,54,55]. Similarly, propolis extract strengthened the growth performance of Nile tilapia [15,18,20] and Mozambique tilapia [21]. The discrepancy in growth performance in response to these herbal extracts may be explained by the fish species, their size, the stocking densities, the culture systems, and most importantly, the source, the composition and the extraction method of these botanical extracts [27,47].

In this study, however, feed intake was significantly reduced and both FCR and PER were improved in PAN-fed fish. Feed efficiency enhancement may be due to the capacity of dietary PAN to improve gut health, promote the secretion of digestive enzymes and facilitate nutrient digestion and absorption by enhancing the beneficial gut microbiota [56,57,58]. The increase in digestive enzyme activity and beneficial gut microbiota in the fish group fed with PAN may support this assumption. Curcumin, which is a major component of PAN, can also support the secretion of other enzymes (intestine alkaline phosphatase, Na+/K+-ATPase and creatine kinase) which play a critical role in nutrient break down and assimilation [9,59]. Curcumin is also identified to enhance the enzymes located at the gut brush border and is well involved in nutrient degradation and absorption [60].

Similar improvements in feed intake and feed efficiency were reported in Nile tilapia [15] and Mozambique tilapia [21] fed propolis- or curcumin-based diets [31,60]. However, curcuminoids exhibit low bioavailability due to their chemical degradation, poor solubility, polarity challenges, susceptibility to autoxidation and limited permeation from the intestine into the portal bloodstream [61]. Meanwhile, some phenolic compounds (such as Artepillin C) have demonstrated the ability to enhance the bioavailability of curcuminoids through in vitro (Caco-2-cell) and in silico approaches [61]. Therefore, the marked increase in feed efficiency observed in this study, especially at 0.5 g kg^−1^, can be attributed to the efficient absorption of these compounds.

The response of farmed aquatic animals to dietary plant extracts is generally evaluated from a biological point of view. So far, no research has considered the economic evaluation of supplemental plant additives, at least for tilapia, which is the target species in this study. Economic analysis of the current results indicated that dietary PAN groups were more profitable than the control, with 0.50 g kg^−1^ providing the highest profit (9.24% increase) compared to the control diet. Therefore, when this product is supplemented at the optimum level, it can lead to significant savings in the feed cost without adversely affecting the fish yield.

As mentioned above, these herbal extracts contain many bioactive compounds. For example, green propolis contains bioactive components such as Artepillin C and flavonoids such as galangin, chrysin, apigenin and so on [16]. Artepillin C has many biological functions, including antioxidant, immunomodulation and metabolic enzyme-modulator, in addition to suppressing the genotoxicity of chemicals [62]. Other flavonoids are also strong antioxidant and anti-inflammatory agents and can treat different degenerative disorders [63]. The other compounds, such as terpenoids, esters and aromatic acids and other derivatives contained in green propolis are also responsible for the antimicrobial and anti-inflammatory properties of propolis [64,65].

Similarly, turmeric contains mainly curcuminoids and essential oils (mainly tumerone) [66]. Curcuminoids comprise three principal compounds; curcumin, desmethoxycurcumin and bisdemethoxycurcumin, at a ratio of about 100:21:3 [47,67]. Turmeric extract also contains high concentrations of polyphenols and flavonoids [59,68]. Phenolic compounds, naturally occurring plant metabolites, have the ability to chelate metallic ions, neutralize free radicals resulting from oxidative stress, and improve antimicrobial activities. However, the antioxidant effect of turmeric depends on the derivatives used and the extraction methods. For example, curcuminoids exhibit higher antioxidant activity than curcumin, desmethoxycurcumin and bisdemethoxycurcumin [67]. However, hydrogenated derivatives of curcumin were demonstrated to show a stronger affinity toward lipid peroxidation and red blood cell hemolysis than curcumin [69]. This means that turmeric and its bioactive constituents have major bioactivities related to human and animal health, including antioxidant, anti-inflammatory and immunomodulatory properties [66].

In the present study, PAN significantly stimulated immune response parameters, including lysozyme activity, alternative complement activity, phagocytic activity, and phenoloxidase activity. PAN also significantly enhanced the antioxidant enzymatic capacity, mainly malondialdehyde, superoxide dismutase-, glutathione peroxidase and catalase. This blend also improved hepatic enzyme activity, gene expression, gut microbiota and gut histology in Nile tilapia fed increasing levels of PAN. Similar findings were observed in Nile tilapia [15,19,20,29], Mozambique tilapia [21,31] and red tilapia (*Oreochromis* sp.) [3]. In these studies, supplemental green propolis and turmeric separately significantly improved immune and antioxidant responses and alleviated oxidative stress.

Liver enzymes (LDH, ALT and AST) are critical biomarkers of hepatic activity and are generally secreted in response to hepatotoxicity, stress or liver malfunction [70]. In this study, groups supplemented with PAN at optimum concentrations reduced the activities of hepatic enzymes in Nile tilapia compared to the control group. This result suggests that PAN extract may contain hepatoprotective components that may enhance the health status of the fish. For example, common propolis contains cinnamic acid, tectochrysin and pinocembrin which play hepatoprotective roles [22,71]. Therefore, dietary propolis decreased serum alanine transferase, aspartate transferase and lactate dehydrogenase values in Mozambique tilapia [21]. Also, [72] reported that dietary curcumin induced hepatoprotective effects in Jian carp (*Cyprinus carpio*) by increasing the antioxidative responses and modulating *IL-1β*, *NF-kB*, *TNF-α* and *IL-12* gene expression. Similarly, curcumin’s hepatoprotective effect in Nile tilapia challenged with aflatoxin B1, as was reported by [73]. Dietary curcumin also alleviated liver damage caused by deltamethrin in the snakehead (*Channa argus*) via *NF-kB* and *Nrf2* signaling pathways [74].

Cytokines are proteins secreted by the immune cells to modulate inflammatory reactions [75]. In the present study, Nile tilapia fed with PAN demonstrated significant upregulation in pro-inflammatory cytokine genes (*IFN-γ*, *TFN-α*, *IL-1β*, *IL-12* and *TGF-β*) compared to fish fed with the control diet. However, gene upregulation leveled off beyond the optimum dietary PAN level (0.50 g kg^−1^) except for *IL-1β*, which was significantly higher at 1.0 g kg^−1^ than at other PAN levels. This finding confirmed the above results and suggested that the dosage of PAN supplementation is important for bioactivity responses. Artepillin C is primarily known for modulating pro-inflammatory markers such as *IL1β* [76] and, probably in the absence of acute infection or stressful conditions, upregulation of some markers, such as *IL-1β*, may occur. Similar results were reported by [55] who found that Nile tilapia fed with curcumin stimulated pro-inflammatory cytokine expression mainly s TNFα and apoptosis markers. The authors of [57] also found that 0.5% of curcumin inclusion in Mozambique tilapia feed significantly upregulated the expression of *IGF-1* and *IGF-2* genes in fish muscle.

Intestinal microbiota contributes to the physiological, immunological and metabolic balance of the host animal [77]. They can partially metabolize ingested food and convert it into microbial biomass and metabolites that contribute to the host’s nutrition [77]. The beneficial bacteria usually coexist in a certain balance with pathogenic microorganisms (*Staphylococccus*, *Streptococcus*, *Salmonella* and *Enterobacter*). Gut microbiota balance is critical for preventing pathogens from proliferating in the gut and replacing beneficial bacteria.

The botanical compounds have been reported to modulate the bacterial communities in fish guts, increase the load of beneficial bacteria and, at the same time, decrease the presence of pathogenic bacteria. For example, [30] demonstrated that Nile tilapia fed diets supplemented with curcumin showed lower counts of intestinal pathogenic bacteria and higher counts of beneficial microbiota (*Bacillus* sp.) than fish fed with the control diet. Similarly, [78] found that the total bacterial counts (TBCs) and total *Lactobacillus* count in Nile tilapia intestines were significantly higher in fish fed turmeric at an optimum level (2 g kg^−1^) than in the control group. Increasing dietary turmeric to 8 g kg^−1^ resulted in an adverse effect on bacterial counts. Along the same line, the total *Vibrio* sp. counts decreased significantly in white-leg shrimp *Liptopenaeus vannamei* fed with curcumin and turmeric [79]. These results suggest that curcumin can positively affect the microbiota balance, but its mode of action should be investigated.

The present study confirmed the above findings and revealed that supplemental PAN significantly increased both the TBC and beneficial bacterial count (*Bacillus* and *Lactobacillus*) and reduced the pathogenic bacterial load (mainly *Pseudomonas*, *Vibrio* and *Salmonella*). This suggests that PAN can constitute a viable strategy for enhancing beneficial gut microbiota and excluding potentially pathogenic bacteria. The observed green propolis effect and upregulation of liver cytokines in the present study may illustrate the immunity modulation that indirectly affects the gut microbiota.

In addition, these results demonstrated that dietary PAN significantly impacted the intestinal histology of Nile tilapia. PAN supplementation increased the intestinal fold length and the number of goblet cells. It was also observed that PAN decreased the interfold space in the anterior, mid and posterior guts. This finding suggests that PAN enhanced nutrient digestion and absorption and feed utilization efficiency as well. The increase in the intestinal fold length of Nile tilapia fed with PAN may improve the surface area for nutrient absorption by increasing both enterocyte proliferation and intestinal mucus secretion (mucins). Mucins are a key factor in lubricating the passage of food and involvement in cell signaling pathways. Moreover, mucins support the immune system by preventing pathogen invasion and protecting epithelial cells from toxins and stressors [80]. The authors of [78] reported similar findings in which supplemented turmeric at 2 g kg^−1^ in feed increased fold length in Nile tilapia.

In the present study, supplemental PAN above 0.50 g kg^−1^ feed levels caused significant retardation or leveling off in all measured parameters. This finding may raise concerns about excessive dietary herbal extract inclusion in diet which may lead to negative effects on fish performance, immune system, antioxidant capacity, and overall health status [21,27,49,60]. For example, it has been suggested that excessive amounts of dietary propolis can result in negative effects on fish, presumably due to the assimilation of excess phenolic compounds, which may, in turn, decrease fish appetite and growth [81]. Similarly, higher inclusion of curcumin resulted in lower growth rates and immune response because curcumin can lead to adverse effects on the digestive tract and provoke inflammatory responses in the intestines [27]. Furthermore, the high inclusion rate of *Tribulus terrestris* in Nile tilapia diets resulted in a significant reduction in fish growth, feed efficiency, lysozyme activity and myeloperoxidase activity [82]. In addition, excessive doses of Chinese medical herb (*Scutellaria radix*) inhibited phagocytosis and respiratory burst activity in Nile tilapia [83]. The authors of [84] also found that supplementing Nile tilapia diets with *Moringa oleifera* aqueous extract beyond the optimum level led to adverse effects on fish health and reproductive performance. These findings may explain the reason behind the reduction or leveling off of the immune response, antioxidant activities, enzyme activities, cytokines upregulation, gut microbiota and gut health at PAN concentrations exceeding the optimum level in the present study (0.5 g kg^−1^).

It should also be mentioned that the levels of PAN that supported optimum response in this study are lower than those reported in Pacific White shrimp (*Litopenaeus vannamei*) feeds. The authors of [17] reported that 1.0 g kg^−1^ of PAN was required for maximum growth performance, immune responses and survival in shrimps challenged against White Spot Syndrome Virus. In addition, much higher levels of active compounds (propolis and curcumin) were required for optimum fish performance when they were used separately in tilapia feeds. Dietary propolis inclusion at about 2–10 g kg^−1^ was required for optimum response of tilapia [15,21,22] while curcumin at 2–30 g kg^−1^ was recommended for their optimum performance [28,31]. Higher levels of dietary curcumin (2–3%) were required for improving growth performance, feed efficiency and immune response of other species, including Gilthead seabream (*Sparus aurata*) [85] and rainbow trout (*Oncorhynchus mykiss*) [86]. Moreover, 3% propolis extract improved digestive enzyme activities, immune responses and antioxidant capacity of beluga (*Sturgeon huso*) [14]. In addition, 3.68 g kg^−1^ of propolis extract supported growth performance of gilthead seabream [87].

The lower PAN levels required for optimum performance of Nile tilapia in the present study could be attributed to the higher concentrations of the active compounds in PAN than those used in these studies. In support, Brazilian green propolis extract contains high concentrations of major active compounds such as Artepillin C, Baccharin and Galangin. Curcuminoids, which are the main bioactive compound in turmeric, are one of the key components in PAN. In addition, the synergistic effects of the key molecules present in PAN may have reduced the amount of PAN needed for optimum performance. Also, the active compounds in this blend may have been more effective than the individual products, as already shown in white-leg shrimp [17]. However, more research is needed to elucidate this assumption.

## 5. Conclusions

This study revealed that supplemental Phyto AquaNity (PAN) significantly improved feed efficiency, immune response and antioxidant capacity of Nile tilapia due to the combined biomolecules Artepillin C and curcuminoids. Additionally, this plant-based mixture significantly enhanced digestive enzyme activity, liver function enzyme activity, cytokine gene expression and microbial communities; thereby improving gut health and overall fish well-being. Supplementing commercial tilapia feed with 0.60–0.85 g PAN kg^−1^ can increase the profitability by up to 9% over the control diet. However, further research is needed to explore the efficacy of this product on other species (e.g., marine species) and its impact on the life cycle assessment of fish farming.

## Figures and Tables

**Table 1:** Ingredients and proximate composition of the experimental diets.

Ingredient	PAN Level (g kg^−1^)			
	0 (C)	0.25	0.50	1.00
Local fishmeal (54% cp)	30.00	30.00	30.00	30.00
Poultry by-product meal (54% cp)	75.00	75.00	75.00	75.00
Soybean meal (46% cp)	425.00	425.00	425.00	425.00
Wheat bran	287.00	287.00	287.00	287.00
Rice bran	115.00	115.00	115.00	115.00
Corn	51.15	50.90	50.65	50.15
Soybean oil	6.00	6.00	6.00	6.00
Monocalcium phosphate	4.00	4.00	4.00	4.00
Sodium bicarbonate	1.00	1.00	1.00	1.00
Calcium carbonate	1.00	1.00	1.00	1.00
Vitamin ^a^ and mineral premix ^b^	2.00	2.00	2.00	2.00
Vitamin C	0.50	0.50	0.50	0.50
Methionine	0.50	0.50	0.50	0.50
Lysine	0.50	0.50	0.50	0.50
Anti-toxin	1.00	1.00	1.00	1.00
Emulsifier	0.25	0.25	0.25	0.25
Phytase enzyme (mL kg^−1^)	0.125	0.125	0.125	0.125
Phyto AquaNity (PAN)	0.00	0.25	0.50	1.00
Proximate analysis				
Moisture	7.80	8.50	8.70	8.90
Crude protein	30.50	30.10	30.00	30.30
Crude lipid	5.60	5.41	5.92	5.58
Ash	8.40	7.8	8.1	7.80
Crude fiber	4.00	4.20	4.00	4.10
NFE ^c^	43.70	44.20	43.40	43.40
Gross energy (MJ kg^−1^) ^d^	17.10	17.02	17.05	16.98

^a^ Vitamin IU or mg kg^−1^: Vit A: 6,000,000 IU, Vit D3: 400,000 IU, Vit E: 60,000, Vit K3: 4000, Vit B1: 4000, Vit B2: 4000, Vit B3: 30,000, Vit B5: 10,000, Vit B6: 3000, Vit B12: 20, Biotin: 75, folic acid: 1500. ^b^ Mineral premix: manganese: 7000, iron: 20,000, zinc: 30,000, copper: 5000, cobalt: 150 and selenium: 1000. ^c^ Nitrogen-free extract (NFE) = 100 − (moisture + crude protein + crude lipid + ash). ^d^ Gross energy, calculated based on 23.64, 39.54 and 17.57 KJ g^−1^ for protein, lipids and carbohydrates, respectively.

**Table 2:** List of primers used for real-time PCR; interleukin 12 (IL-12); interleukin-1 beta (IL-1β); tumor necrosis factor alpha (TNF-α); transforming growth factor (TGF-β); interferon-gamma (IFN-γ); interleukin-4 (IL-4).

Gene	Forward Sequence (5′ → 3′)	Accession No	Amplification Efficiency (%)	Product Length (bp)
*IL-12*	F (5′ → 3′): GGGTGCGAGTCAGCTATGAG R (5′ → 3′): GGTTGTGGATTGGTTGCGTC	XM_003437924.4	99.1	159
*IL-1β*	F (5′ → 3′): GACACTGCTTCTGAACTACAAGT R (5′ → 3′): TCAGCACTGGCTCTGAAGTG	XM_019365844.2	98.4	209
*TNF-α*	F (5′ → 3′): GCAGCTGAATGAACCTCTCAC R (5′ → 3′): GTTCTCAGTCTGTCCCCAGC	XM_013266976.3	98.7	760
*TGF-1β*	F (5′ → 3′): GTCCTGCAAGTGCAGCTAGA R (5′ → 3′): CATGCCTGTGTGAAACGACTG	XM_005463992.4	99.4	137
*IFN-γ*	F (5′ → 3′): GGGTGGTGTTTTGGAGTCGT R (5′ → 3′): CATCTGTGCCTGGTAGCGAG	XM_013266976.3	97.7	161
*IL-4*	F (5′ → 3′): CAGCGAGAGAGAACTCGTGC R (5′ → 3′): GGTTTCCTTCTCCGTCGTGT	NM_214123.1	98.3	80
*CAT*	F (5′ → 3′): TCCTGAATGAGGAGGAGCGA R (5′ → 3′): ATCTTAGATGAGGCGGTGATG	XM_003447521.5	97.5	129
*β* *-Actin*	F: ACAACTCAGGCGCAGAGAAT R: TCCTGAGTCAAGCGCCAAAA	XM_003443127.5	99.7	73

**Table 3:** Growth performance, feed utilization and profitability (mean ± SE; n = 3) of Nile tilapia fed with the experimental diets. Values with different superscripts in the same row are significantly different (*p* < 0.05).

Parameter	PAN_0_ (C)	PAN_0.25_	PAN_0.5_	PAN_1_
Growth and feed efficiency				
Wi (g fish^−1^)	74.86 ± 1.27	74.55 ± 0.82	73.59 ± 1.10	73.88 ± 0.67
Wf (g fish^−1^)	186.10 ± 2.29 ^a^	184.85 ± 2.02 ^a^	190.72 ± 4.09 ^a^	187.48 ± 3.39 ^a^
WG (g fish^−1^)	111.24 ± 3.27 ^a^	110.30 ± 2.18 ^a^	117.13 ± 5.21 ^a^	112.60 ± 6.94 ^a^
WG (%)	148.68 ± 6.17 ^a^	147.95 ± 1.45 ^a^	159.11 ± 3.14 ^a^	152.54 ± 11.61 ^a^
SGR (% day^−1^) *	1.15 ± 0.02 ^a^	1.12 ± 0.01 ^a^	1.19 ± 0.02 ^a^	1.15 ± 0.06 ^a^
FI (g fish^−1^)	161.76 ± 3.51 ^a^	149.29 ± 1.78 ^b^	143.05 ± 2.35 ^b^	147.46 ± 3.29 ^b^
FCR *	1.46 ± 0.03 ^a^	1.35 ± 0.01 ^b^	1.24 ± 0.03 ^c^	1.31 ± 0.05 ^b,c^
PER *	2.25 ± 0.05 ^a^	2.45 ± 0.02 ^a,b^	2.69 ± 0.08 ^b^	2.53 + 0.20 ^b^
Economic analysis				
Fish production (kg m^−1^)	3.72 ± 0.05 ^a^	3.70 ± 0.04 ^a^	3.75 ± 0.02 ^a^	3.73 ± 0.07 ^a^
Fish sale (USD m^−1^)	7.82 ± 0.10 ^a^	7.76 ± 0.08 ^a^	7.87 ± 0.05 ^a^	7.83 ± 0.14 ^a^
Gross profit (USD m^−1^)	4.58 ± 0.10 ^a^	4.76 ± 0.07 ^a^	5.00 ± 0.05 ^b^	4.84 ± 0.11 ^b,a^
Profitability (%) over the control diet		4.08 ± 1.46^a^	9.24 ± 1.13 ^b^	5.67 ± 2.29 ^a^

* Wi, initial weight; Wf, final weight; WG, weight gain; SGR, specific growth rate; FI, feed intake; FCR, feed conversion ratio; PER, protein efficiency ratio.

**Table 4:** Proximate analysis of Nile tilapia flesh (mean ± SE; n = 3) fed the tested diets (on wet weighted basis). Values with different superscripts in the same row are significantly different (*p* < 0.05).

Parameter (%)	Initial	PAN_0_ (C)	PAN0_.25_	PAN_0.5_	PAN_1_
Moisture	75.13	74.57 ± 0.25	74.30 ± 1.39	75.21 ± 0.39	74.70 ± 0.48
Crude protein	18.96	21.38 ± 0.45	21.51 ± 1.56	20.71 ± 0.51	20.78 ± 0.11
Crude lipid	3.09	2.45 ± 0.26	2.67 ± 0.26	2.63 ± 0.41	3.04 ± 0.54
Ash	1.81	1.49 ± 0.09	1.47 ± 0.20	1.41 ± 0.07	1.49 ± 0.10

**Table 5:** Digestive and hepatic enzyme activity (mean ± SE; n = 3) of Nile tilapia fed the tested diets. Values with different superscripts in the same row are significantly different (*p* < 0.05).

Enzymes	PAN_0_ (C)	PAN_0.25_	PAN_0.5_	PAN_1_
Amylase (U mg^−1^)	21.58 ± 2.36 ^a^	27.10 ± 1.95 ^b^	38.02 ± 2.02 ^c^	40.58 ± 0.89 ^c^
Lipase (U mg^−1^)	50.12 ± 2.44 ^a^	55.29 ± 3.46 ^a^	72.21 ± 3.66 ^b^	73.39 ± 3.73 ^b^
Protease (U mg^−1^)	62.18 ± 2.74 ^a^	71.69 ± 3.10 ^b^	85.10 ± 2.34 ^c^	87.26 ± 3.66 ^c^
AST (U L^−1^)	16.67 ± 2.08 ^a^	13.33 ± 1.00 ^a,b^	10.33 ± 0.57 ^b^	12.33 ± 0.57 ^b^
ALT (U L^−1^)	10.67 ± 1.15 ^a^	8.00 ± 1.00 ^a,b^	5.66 ± 0.57 ^b^	7.33 ± 1.15 ^b^
LDH (U L^−1^)	135.48 ± 4.52 ^a^	129.94 ± 0.50 ^a,b^	123.77 ± 2.20 ^b^	125.82 ± 2.29 ^b^

**Table 6:** Immune response and antioxidant capacity (mean ± SE; n = 3) of Nile tilapia fed the test diets. Values with different superscripts in the same row are significantly different (*p* < 0.05).

Parameter	PAN_0_ (C)	PAN_0.25_	PAN_0.5_	PAN_1_
PA (µM mL^−1^)	1.22 ± 0.04 ^a^	1.26 ± 0.03 ^a^	1.47 ± 0.09 ^b^	1.51 ± 0.05 ^b^
PO (mU mL^−1^)	98.63 ± 2.13 ^a^	104.4 ± 2.26 ^a,b^	111.0 ± 2.27 ^b,c^	111.3 ± 1.33 ^c^
ACH50 (ng mL^−1^)	44.16 ± 2.62 ^a^	50.25 ± 1.05 ^b^	56.58 ± 2.49 ^c^	60.19 ± 2.63 ^c^
LSZ (ng mL^−1^)	82.57 ± 0.55 ^a^	87.85 ± 1.59 ^b^	101.30 ± 2.76 ^c^	104.9 ± 2.16 ^c^
SOD (mU mL^−1^)	29.93 ± 2.06 ^a^	31.42 ± 0.95 ^a^	39.64 ± 1.41 ^b^	39.62 ± 2.00 ^b^
MDA (mU mL^−1^)	23.41 ± 2.51 ^a^	17.13 ± 1.75 ^b^	14.14 ± 1.07 ^b^	13.41 ± 1.87 ^b^
GPx (mU mL^−1^)	3.47 ± 0.12 ^a^	4.35 ± 0.09 ^b^	4.51 ± 0.07 ^b^	4.55 ± 0.15 ^b^
Catalase	447.57 ± 12.39 ^a^	520.30 ± 8.80 ^b^	530.90 ± 8.61 ^b^	535.57 ± 4.40 ^b^

**Table 7:** Liver gene expression of Nile tilapia fed the experimental diets (mean ± SE; n = 3). Values with different superscripts in the same row are significantly different (*p* < 0.05).

Gene	PAN_0_ (C)	PAN_0.25_	PAN_0.5_	PAN_1_
*IFN-γ* (pg mL^−1^)	128.23 ± 1.16 ^a^	132.57 ± 0.55 ^b^	137.0 ± 1.21 ^c^	136.57 ± 0.47 ^c^
*TNF-α* (copies mL^−1^)	338.47 ± 1.92 ^a^	342.9 ± 0.8 ^b^	348.83 ± 0.76 ^c^	350.1 ± 2.45 ^c^
*TGF-1β* (pg mL^−1^)	65.60 ± 1.14 ^a^	66.63 ± 0.55 ^a,b^	69.83 ± 1.13 ^b,c^	72.07 ± 1.63 ^c^
*IL-1β* (copies mL^−1^)	563.57 ± 1.32 ^a^	569.70 ± 0.45 ^b^	575.03 ± 1.51 ^c^	582.57 ± 2.17 ^d^
*IL-4* (copies mL^−1^)	81.33 ± 1.52 ^a^	88.33 ± 1.52 ^b^	96.67 ± 1.52 ^c^	100.67 ± 1.15 ^c^
*IL-12* (copies mL^−1^)	244.33 ± 1.15 ^a^	258.67 ± 6.03 ^b^	276.67 ± 2.08 ^c^	279.00 ± 1.73 ^c^
CAT (ng g^−1^)	379.97 ± 0.66 ^a^	382.87 ± 1.07 ^b^	383.0 ± 0.66 ^b^	383.53 ± 0.41 ^b^

IFN-γ, interferon-gamma; TNF-α, tumor necrosis factor alpha; TGF-1β, transforming growth factor-1 beta; IL-1β, interleukin-1 beta; IL-4, interleukin-4; IL-12, interleukin 12; CAT, chloramphenicol acetyltransferase.

**Table 8:** Gut bacterial counts (CFU mL^−1^) of Nile tilapia fed the test diets (mean ± SE; n = 3). Values with different superscripts in the same row are significantly different (*p* < 0.05).

Treatment	PAN_0_	PAN_0.25_	PAN _0.50_	PAN_1_
Total bacterial counts	142.67 ± 5.37 ^a^	185.67 ± 6.03 ^b^	217.00 ± 4.42 ^c^	206.33 ± 2.97 ^d^
Beneficial bacterial counts	44.33 ± 4.04 ^a^	112.3 ± 10.78 ^b^	158.3 ± 5.68 ^c^	142.0 ± 2.64 ^d^
Total pathogenic bacterial counts	98.33 ± 7.63 ^a^	73.33 ± 6.11 ^b^	58.33 ± 6.50 ^b,c^	64.33 ± 5.85 ^b,c^

**Table 9:** Histological parameters (mean ± SE; n = 3) of Nile tilapia fed the experimental diets. Values with different superscripts in the same row are significantly different (*p* < 0.05).

Parameters	PAN_0_ (C)	PAN_0.25_	PAN_0.5_	PAN_1_
Anterior gut:				
Intestinal fold length (µm)	133.21 ± 9.75 ^a^	138.20 ± 13.56 ^a^	175.0 ± 12.02 ^b^	197.0 ± 8.57 ^b^
Interfold space (µm)	80.40 ± 1.36 ^a^	73.83 ± 5.74 ^a^	68.45 ± 6.10 ^a,b^	60.13 ± 6.93 ^b^
Goblet cells number (mm^−2^)	51.99 ± 3.50 ^a^	57.91 ± 4.73 ^a,b^	67.19 ± 3.62 ^b,c^	72.84 ± 3.76 ^c^
Middle gut				
Intestinal fold length (µm)	155.4 ± 13.12 ^a^	203.0 ± 13.47 ^b^	284.5 ± 10.99 ^c^	305.5 ± 15.58 ^c^
Interfold space (µm)	61.00 ± 5.74 ^a^	40.04 ± 2.73 ^b^	36.59 ± 5.62 ^b^	32.62 ± 6.62 ^b^
Goblet cells number (mm^−2^)	77.62 ± 6.20 ^a^	88.98 ± 3.80 ^a,b^	101.43 ± 6.44 ^b^	107.1 ± 7.56 ^b^
Posterior gut				
Intestinal fold length (µm)	80.60 ± 5.76 ^a^	99.55 ± 2.09 ^a^	126.5 ± 7.41 ^b^	132.1 ± 8.99 ^b^
Interfold space (µm)	126.00 ± 24.61 ^a^	90.95 ± 2.08 ^b^	82.80 ± 1.98 ^b^	74.62 ± 8.12 ^b^
Goblet cells number (mm^−2^)	28.27 ± 2.31 ^a^	46.93 ± 9.95 ^b^	53.18 ± 8.28 ^b^	55.29 ± 4.53 ^b^

## Data Availability

Data from this study are available upon request.
